# Supplementary material for: Accessing Occupational Health Services in the Southern African Development Community Region
Source: Int J Environ Res Public Health. 2020 Sep 17;17(18):6767. doi: 10.3390/ijerph17186767 (PMC7559743; doi:10.3390/ijerph17186767)
Supplement: Supplementary File 1 [file ijerph-17-06767-s001.pdf]

**Occupational health services provision at primary health facilities (Zambia 2020)**

**Part A: Demographic Data**

1. Industry Type: (please tick)

Mining ☐ Hospital ☐ Clinic ☐ Center of excellent ☐

Other – Specify____________________________________________________

2. Employee complement: <100 ☐ 100 – 699 ☐ >700 ☐
3. Nurses complement:

   With Occupational Health (OH) Qualifications_______ without OH qualifications_________

4. Your Job position:__________________________________________________

5. Your academic Qualifications
   1.____________________________________________________________
   2. ____________________________________________________________
   3.____________________________________________________________
   4.____________________________________________________________

**PART B: Occupational health services provided by clinics/health facilities**

1. **Services offered at your company clinic/health facility**
   Please tick the appropriate box

| | Yes | No | Onsite | Outsourced |
|---|---|---|---|---|
| | | | | |
| Primary health services (communicable and non-communicable diseases) | | | | |
| HIV and TB services | | | | |
| Health Risk Assessment (hazard Identification and Risk Assessment) | | | | |
| Medical Surveillance Programme (see also below) | | | | |
| Pre- employment/Pre-placement medicals | | | | |
| Periodic Medicals | | | | |
| Exit medicals | | | | |
| Pneumoconiosis (Silicosis in co-workers pneumoconiosis) | | | | |
| Audiometry | | | | |
| Spirometry | | | | |
| Snellen Chart vision test | | | | |
| Vision testing other than Snellen Chart | | | | |
| Alcohol testing | | | | |
| Health promotion at work stations | | | | |
| Biological effects monitoring (blood or urine tests) e.g. Blood lead levels | | | | |
| Rehabilitation – physiotherapy etc | | | | |
| Others (specify) | | | | |

**PART C: Training Needs**

1. State the occupational health areas/topics that you and your team require training/development in for you to be able to offer good occupational health services at your organisation.

   ________________________________________________________________________
   ________________________________________________________________________
   ________________________________________________________________________
   ________________________________________________________________________
   ________________________________________________________________________
   ________________________________________________________________________
   ________________________________________________________________________
   ________________________________________________________________________
